# Neutrophil Extracellular Traps as an Adhesion Substrate for Different Tumor Cells Expressing RGD-Binding Integrins

**DOI:** 10.3390/ijms19082350

**Published:** 2018-08-09

**Authors:** Marcello Monti, Viviana De Rosa, Francesca Iommelli, Maria Vincenza Carriero, Cristina Terlizzi, Rosa Camerlingo, Stefania Belli, Rosa Fonti, Giovanni Di Minno, Silvana Del Vecchio

**Affiliations:** 1 Dipartimento di Medicina Clinica e Chirurgia, Università degli Studi di Napoli “Federico II”, Via S. Pansini 5, 80131 Naples, Italy; marcello.monti@unina.it (M.M.); diminno@unina.it (G.D.M.); 2 Istituto di Biostrutture e Bioimmagini, Consiglio Nazionale delle Ricerche, 80145 Naples, Italy; viviana.derosa@ibb.cnr.it (V.D.R.); francesca.iommelli@ibb.cnr.it (F.I.); rosa.fonti@ibb.cnr.it (R.F.); 3 Dipartimento di Oncologia Sperimentale, IRCCS Istituto Nazionale Tumori “Fondazione G. Pascale”, 80145 Naples, Italy; m.carriero@istitutotumori.na.it (M.V.C.); r.camerlingo@istitutotumori.na.it (R.C.); 4 Dipartimento di Scienze Biomediche Avanzate, Università degli Studi di Napoli “Federico II”, Via S. Pansini 5, 80145 Naples, Italy; cr.terlizzi88@gmail.com; 5 Istituto di Genetica e Biofisica, Consiglio Nazionale delle Ricerche, 80131 Naples, Italy; stefania.belli@igb.cnr.it

**Keywords:** neutrophil extracellular traps, cell adhesion, integrins, fibronectin, cancer metastasis

## Abstract

Neutrophil extracellular traps (NETs), in addition to their function as a host defense mechanism, play a relevant role in thrombus formation and metastatic dissemination of cancer cells. Here we screened different cancer cell lines endogenously expressing a variety of integrins for their ability to bind to NETs. To this end, we used NETs isolated from neutrophil-like cells as a substrate for adhesion assays of HT1080, U-87 MG, H1975, DU 145, PC-3 and A-431 cells. Levels of α5, αIIb, αv, β1, β3 and β5 chains were determined by western blot analysis in all cell lines and levels of whole integrins on the plasma membrane were assessed by fluorescence-activated cell sorting (FACS) analysis. We found that high levels of α5β1, αvβ3 and αvβ5 enhance cell adhesion to NETs, whereas low expression of α5β1 prevents cell attachment to NETs. Excess of cyclic RGD peptide inhibited cell adhesion to NETs by competing with fibronectin within NETs. The maximal reduction of such adhesion was similar to that obtained by DNase 1 treatment causing DNA degradation. Our findings indicate that NETs from neutrophil-like cells may be used as a substrate for large screening of the adhesion properties of cancer cells expressing a variety of RGD-binding integrins.

## 1. Introduction

Neutrophil extracellular traps (NETs) are web-like structures released from activated neutrophils exposed to different pathogens including bacteria, viruses or fungi [1]. They are composed of decondensed chromatin associated with histones and other granular proteins such as myeloperoxidase (MPO) and neutrophil elastase (NE) [2]. Once outside the cell, their main function is the entrapment of pathogens and inactivation of virulent factors through enzymatic degradation [3,4].

In response to inflammatory stimuli, neutrophils migrate from the blood stream to the site of infection where, in addition to the well-known mechanisms of phagocytosis and degranulation, they can extrude NETs with a multistep process termed netosis [5,6,7]. This process is characterized by morphological changes of polymorphonucleocytes (PMN), including chromatin decondensation and loss of the classical lobulated nuclear morphology, disruption of nuclear membrane and subsequent mixing of nuclear and cytoplasmic content followed by extrusion into the extracellular environment through plasma membrane disintegration [8,9]. Furthermore, several biochemical changes occur during netosis, including activation of peptidylarginine deiminase 4 (PAD4) [10], an enzyme that induces citrullination of histones, along with increased activity of MPO [11,12] and NE [13]. Due to their critical role in netosis, citrullinated histones, MPO and NE are considered the major biomarkers of NETs along with extracellular double-stranded DNA. In a recent study, we found that in addition to citrullinated histones, MPO and NE, NETs contain fibronectin, a well-known substrate of several integrins, secreted by stimulated neutrophil-like cells [14]. Adhesion of human K562 cells, differentially expressing α5β1 and αvβ3 integrins, to NETs was inhibited by both degradation of DNA and blocking antibodies against those integrins.

Moreover, consistent evidence indicated that NETs may have an additional role in cancer-associated thrombosis [15,16,17] and the dissemination of cancer cells [18,19]. In particular, Demers et al. [16] demonstrated that neutrophils from tumor-bearing animals are more prone to release NETs, thus generating a pro-coagulant and pro-thrombotic state in these animals. Interestingly, NETs have been found in tumors at sites of neutrophil infiltration where they may influence the cancer microenvironment, favoring local invasion [16]. Furthermore, previous studies in a model of systemic infection showed that circulating tumor cells become trapped within NETs in lung capillaries or hepatic sinusoidal spaces [18].

The aim of the present study was to test whether NETs can be an adhesion substrate for different tumor cells expressing RGD-binding integrins, and whether the levels of expression of these integrins may affect cell adhesion to NETs. The tripeptide RGD (Arg-Gly-Asp) is the sequence contained in many extracellular matrix components such as fibronectin, vitronectin, and fibrinogen and it serves as a binding site for different integrins [20,21]. Several authors have reported the results of the adhesion of cancer cells to neutrophils grown as a monolayer and stimulated to release NETs [18,22]. However, in these experimental conditions, one cannot avoid cell–cell interactions and contamination with integrin substrates present in blood or on the surface of neutrophils. For these reasons, we decided to perform adhesion assays using isolated NETs as an adhesion substrate and differentiated HL-60 cells as a stable source for their generation.

## 2. Results

**Figure 1:**
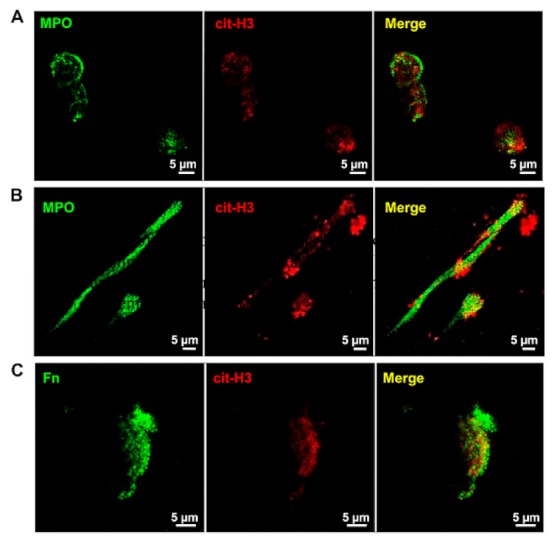
Characterization of selected protein components of neutrophil extracellular traps (NETs). (**A**,**B**) Representative images obtained by confocal microscopy showing co-localization of myeloperoxidase (MPO) (green) and citrullinated histone H3 (cit-H3) (red) in neutrophil-like cells stimulated in serum-free conditions with 25 μM calcium ionophore for 4 h (**A**) or in isolated NETs (**B**); and (**C**) Representative images obtained with confocal microscopy showing co-localization of fibronectin (green) and cit-H3 (red) in isolated NETs. Scale bar 5 μm.

Human HL-60 cells were differentiated into neutrophil-like cells as previously described [14] and then treated with calcium ionophore in order to release NETs. Conditioned medium from treated cells was then subjected to centrifugation to obtain cell-free NET-enriched suspensions that were subsequently employed as an adhesion substrate. NET production was preliminarily tested to confirm the presence of extracellular double-stranded DNA and major NET biomarkers such as MPO and citrullinated histone H3 (cit-H3). Figure 1 shows the presence and co-localization of MPO (green) and cit-H3 (red) in stimulated cells (Panel A) and isolated NETs (Panel B) by confocal microscopy. Since we have previously shown the presence of fibronectin in the web-like structure of NETs [14], and this protein is a well-known substrate for integrin-dependent adhesion, we performed co-immunolocalization studies with anti-fibronectin and anti-cit-H3 antibodies in isolated NETs and representative images are showed in Figure 1C.

**Figure 2:**
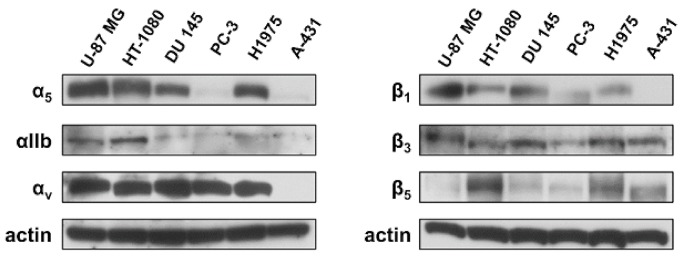
Expression of integrin chains in a panel of cancer cell lines. Whole cell lysates from U-87 MG, HT-1080, DU 145, PC-3, H1975 and A-431 cells were subjected to western blot analysis to determine levels of α5, αIIb, αv, β1, β3 and β5 integrin chains. Actin ensured equal loading.

To investigate the ability of different RGD-binding integrins to mediate adhesion to NETs, a panel of human cancer cell lines with a variable expression of α5β1, αvβ3, αIIbβ3 and αvβ5 integrins was subjected to adhesion assays to NETs in the presence of an excess of cyclic RGD peptide (cRGD) or cyclic control peptide (cCTRL) and compared to samples treated with DNase 1. Diluent or conditioned medium (CM) from unstimulated neutrophil-like cells were used as negative controls. The expression of each integrin chain was tested by western blotting in whole lysates of U-87 MG, HT-1080, DU 145, PC-3, H1975 and A-431 cells. Figure 2 shows representative images of western blot analysis. Levels of α5 were faintly detected in PC-3 and A431 cells, whereas all the other cell lines expressed relatively high levels of this integrin chain. High expression of αv was found in all cell lines except A-431 cells, whereas the levels of the αIIb chain were higher in U-87 MG, HT-1080 and H1975 cells than in DU 145, PC-3 and A-431 cells. Among all β chains analyzed, A-431 cells lacked the β1 chain, whereas PC-3 cells showed only a faint signal for the same protein. Finally, β5 expression was faintly detected in U-87 MG cells, whereas β3 was found in all cell lines. Results of the densitometric analysis of western blotting signal are reported in Figure S1.

To test the possible assembly of these integrin chains on the plasma membranes, fluorescence-activated cell sorting (FACS) analysis was performed in all cell lines, and the results are reported in Table 1. A limited expression of integrin αIIbβ3 (≤10%) was found in all cell lines. The integrin expressed in the highest percentage of HT-1080 cells was αvβ5 (96%), followed by α5β1 (29%). U-87 MG cells showed positive staining for α5β1 and αvβ3 in 86% and 79% of cells, respectively. An equivalent expression of αvβ5 and αvβ3 was found in a high percentage of H1975 cells (80% and 75%, respectively), followed by α5β1 expression in 31%. DU 145 cells expressed mainly α5β1 (78%) and αvβ5 (76%) integrins. Finally, PC-3 and A-431 cells showed positive staining in a considerable percentage of cells only for αvβ5 integrin (74% and 71%, respectively).

**Figure 3:**
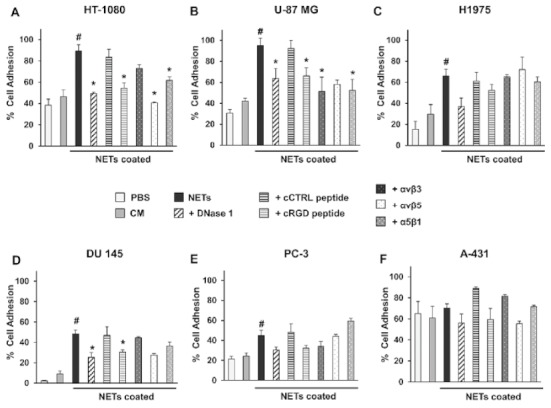
(**A**–**F**) Adhesion of different cancer cell lines to NETs. Isolated NETs were used as an adhesion substrate to coat multi-well plates, whereas phosphate buffered saline (PBS) or conditioned medium (CM) from unstimulated neutrophil-like cells were used as negative controls. Cells were then added to each well in serum-free conditions and allowed to adhere for 1 h at 37 °C in the absence or presence of DNase 1, cyclic control peptide (cCTRL), cyclic RGD peptide (cRGD) and the blocking antibody recognizing the selected integrin. After removal of non-adherent cells and a gentle washing, adherent cells were detached and counted. Results are expressed as percentage of adherent cells compared to the total number of added cells (mean ± SE). Statistical significant differences versus negative controls (PBS and CM) are indicated by the symbol # (*p* < 0.05), whereas versus NETs by the symbol * (*p* < 0.05).

Cell adhesion assays were then performed in all cell lines tested (Figure 3). Compared to negative controls, adhesion to NETs was significantly higher in all cell lines except A-431 cells. Moreover, incubation of all cell lines with the cCTRL peptide did not affect adhesion to NETs. Conversely, the addition of the cRGD peptide reduced adhesion of HT-1080 and U-87 MG cells to values similar to those obtained with DNase 1 treatment (Figure 3A,B). Similar results were obtained by pre-incubating cells with specific antibodies blocking the selected integrin. Analysis of variance followed by pairwise comparisons showed a statistically significant difference in adhesion among multiple experimental conditions in HT-1080 (*f*-ratio = 11.10, *p* < 0.001) and U-87 MG (*f*-ratio = 6.83, *p* < 0.001) cells. In particular, adhesion to NETs was significantly reduced (*p* < 0.05) by the cRGD peptide, DNase 1 treatment and anti-α5β1 antibody in both cell lines, whereas anti-αvβ5 and anti-αvβ3 antibodies significantly reduced adhesion in HT-1080 and U-87 MG cells, respectively. In H1975 cells, competition with the cRGD peptide caused a partial reduction of adhesion to NETs that was lower than that obtained with DNase 1 treatment (Figure 3C), although neither of them achieved statistical significance. Similarly, no significant reduction of cell adhesion was observed with the addition of any of the selected blocking antibodies, despite the expression of considerable levels of αvβ3 and αvβ5. Furthermore, in a parallel experiment, pre-incubation of this cell line with a combination of anti-α5β1, anti-αvβ3 and anti-αvβ5 antibodies did not affect cell adhesion to NETs when compared to the positive control (65% vs. 66%). Therefore, it is likely that other factors or integrins may promote cell adhesion of this cell line to NETs. In DU 145 cells, analysis of variance followed by pairwise comparison showed an equivalent statistically significant reduction of adhesion by both the cRGD peptide (*p* < 0.05) and DNase 1 treatment (*p* < 0.05) that, however, remained significantly higher (*p* < 0.05) than the negative controls (Figure 3D). Despite the adhesion of DU 145 cells was reduced as a result of pre-incubation with anti-αvβ5 and anti-α5β1 antibodies, a statistically significant difference was not achieved. DNase 1 treatment and pre-incubation with the cRGD peptide or any of the selected blocking antibodies did not significantly affect the adhesion of PC3 cells to NETs (Figure 3E). Finally, A-431 cells showed the lowest NET-dependent and integrin-dependent adhesion, with values similar to the negative controls in all conditions (Figure 3F) (*f*-ratio = 1.92, *p* = 0.11).

## 3. Discussion

Our study showed that isolated NETs, obtained from stimulation of neutrophil-like cells, express the same major markers of NETs released from circulating human neutrophils and maintained similar structural features. The advantage to use neutrophil-like cells instead of circulating human neutrophils to produce NETs relies on the fact that neutrophil-like cells are readily available and can provide an abundant source of NETs, allowing for the screening of different tumor cell lines in NET adhesion assays. Previous studies [23] reported a simplified procedure for neutrophil isolation and NET production from the blood of healthy volunteers. However, large volumes of blood samples are required to obtain an adequate amount of cell-free NETs, and many preparations are usually needed to perform a complete set of adhesion assays. Since each preparation derives from a different donor, a large variability affects the results of these experiments [24]. Using neutrophil-like cells as a source of NETs for adhesion assays reduces such experimental variability and allows for the simultaneous screening of different cancer cell lines with a more standardized method.

Our findings indicate that RGD-binding integrins may have a major role in the cell adhesion of different carcinoma cells to NETs, since high levels of α5β1, αvβ3 and αvβ5 in cells enhances adhesion to NETs, whereas low expression of α5β1 prevents cell adhesion to NETs. In a previous study [14], we showed that NETs obtained from neutrophil-like cells contained fibronectin, and that adhesion of K562 cells to NETs is mediated by the α5β1 and αvβ3 integrins through engagement of fibronectin. Here, we tested the adhesion of different cancer cell lines expressing a large spectrum of RGD-binding integrins to NETs and simultaneously compared their adhesion properties. α5β1 seems to play a primary role in adhesion to NETs. To this respect, a recent study reported that silencing of the β1 integrin with targeted siRNA in A549 lung cancer cells caused a decrease in cell adhesion to NETs in liver sinusoids as assessed by intravital microscopy in a mouse model of metastatic dissemination [25]. The maximal reduction of integrin-dependent adhesion to NETs was similar to that obtained after DNase 1 treatment, confirming that both DNA and fibronectin were relevant in determining cell attachment to NETs. It is conceivable that RGD-binding integrins may serve to anchor cells to the web-like structure of NETs, allowing their close interaction with DNA/histone complexes. The disruption of DNA structure or competition for integrin binding to fibronectin equally impairs cell adhesion to NETs.

In conclusion, the screening of different cancer cell lines expressing a variety of RGD-recognizing integrins revealed that cell adhesion to NETs is dependent on the levels of these integrins, which promote a stable cell interaction with DNA by binding to fibronectin. Furthermore, our approach highlighted the possibility of performing a large screening of different cancer cell lines, testing their adhesion properties to isolated NETs while avoiding co-culture with circulating neutrophils from blood donors and the need for large volumes of human blood samples. In this way, circulating cancer cells isolated from patients or tumor cell lines derived from human specimens can be easily tested in vitro, mimicking cancer cell homing at sites of NET deposition. 

## 4. Materials and Methods

### 4.1. Cell Lines and Treatment

Human HL-60 cells can be differentiated into neutrophil-like cells upon treatment with dimethyl sulfoxide (DMSO), and they have been widely used to study several leukocyte functions including oxidative burst, adhesion, chemotaxis and migration [26]. For the present study, HL-60 cells were kindly provided by Stoppelli, et al. [27] and were maintained in Roswell Park Memorial Institute (RPMI) 1640 supplemented with 10% fetal bovine serum (FBS).

Human HL-60 cells were differentiated into neutrophil-like cells using a standard protocol with slight modifications [28]. Briefly, 1 × 10^6^ HL-60 cells were grown in medium with 1.3% DMSO using T25 flasks in a humidified incubator at 5% CO_2_ and 37 °C for 5 days, adding fresh medium with 1.3% DMSO to the flasks on the third day. Then, 1.5 × 10^6^ treated cells were plated into petri dishes containing 10 mL of fresh medium with 1.3% DMSO to complete differentiation and allow cell attachment for an additional two days. Differentiation was confirmed by assessing changes in cell morphology with May-Grunwald-Giemsa staining and testing the expression of CD11b, CD16b and CD177 antigens, markers of neutrophil differentiation, by flow cytometry (BD FACSAria II, Franklin Lakes, NJ, USA) as previously described [14]. The percentage of positively stained cells tested for neutrophil markers was always higher than 60%.

To induce the release of NETs, neutrophil-like cells were treated with 25 µM calcium ionophore (A23187, Sigma-Aldrich, St. Louis, MO, USA). Briefly, differentiated cells were plated into petri dishes in RPMI 1640 at a density of 1 × 10^6^ cells/mL and exposed to calcium ionophore for 4 h in a humidified incubator. After treatment, the conditioned medium was recovered and centrifuged at 310× *g* for 10 min at 4 °C to obtain a cell-free NET-enriched supernatant. This supernatant was then centrifuged at 18,000× *g* for 10 min at 4 °C, and the pellet containing NETs was resuspended in 100 µL of cold PBS. Finally, double-stranded DNA concentration was determined using a NanoDrop ND-1000 spectrophotometer with V3.5.2 software (NanoDrop Technology, Cambridge, UK) and cell-free isolated NETs were used as a stock for further experiments. Different stock suspensions were prepared and used to obtain experimental replicates.

Several tumor cell lines were tested for adhesion using cell-free NET-enriched suspensions as substrate, including: human epidermoid carcinoma A-431, fibrosarcoma HT-1080, glioblastoma U-87 MG, non-small cell lung cancer H1975, prostate cancer DU 145 and PC-3 cells. A-431, HT-1080, U-87 MG, DU 145 and PC-3 cells were grown in Dulbecco’s Modified Eagle Medium (DMEM) (Gibco Life Technologies, Waltham, MA, USA) containing 10% FBS, whereas H1975 cells were grown in complete RPMI 1640 (Gibco Thermo Fisher, Waltham, MA, USA) containing 10% FBS. All cell lines were maintained in a humidified incubator at 5% CO_2_ and 37 °C.

### 4.2. Characterization of NETs by Fluorescence and Confocal Microscopy

NET formation was evaluated by fluorescence microscopy. Neutrophil-like cells (5 × 10^5^) were added to 24-well tissue culture plates with glass coverslips in serum-free Hanks’ balanced salt solution (HBSS) with calcium and magnesium chloride for 1 h at 37 °C with 5% CO_2_. Then, cells were stimulated with calcium ionophore and stained with 5 µM Sytox Green cell-impermeable nucleic acid dye (Invitrogen, Carlsbad, CA, USA). Each coverslip was then washed in PBS, mounted on a glass slide and examined by fluorescence microscopy.

Glass coverslips were incubated overnight at 4 °C with 5 µg of isolated NETs and then subjected to co-immunolocalization study using confocal microscopy (510 META LSM, Carl Zeiss, Oberkochen, Germany). Rabbit polyclonal antibodies recognizing citrullinated histone H3 (10 µg/mL, ab5103, Abcam, Cambridge, UK) and mouse monoclonal antibody recognizing myeloperoxidase (MPO) (10 µg/mL, clone 2C7, ab25989, Abcam) were employed in co-immunolocalization studies. After several washes with PBS containing 1% bovine serum albumin (BSA), 1:700 goat Alexa Fluor 594 anti-rabbit IgG and 1:500 rabbit Alexa Fluor 488 anti-mouse IgG (Molecular Probes, Eugene, OR, USA) were added for 45 min at room temperature in the dark. Then, glass coverslips were washed twice with PBS, mounted with ProLong Gold Antifade Reagent (Invitrogen) and examined by confocal microscopy. In parallel experiments, co-immunolocalization studies were performed in neutrophil-like cells stimulated to release NETs by calcium ionophore as described above.

### 4.3. FACS Analysis of Integrin Expression

Levels of αvβ3, αvβ5, αIIbβ3 and α5β1 integrins were determined by fluorescence-activated cell sorting (BD FACSAria II, Franklin Lakes, NJ, USA) [29]. Briefly, selected cell lines (5 × 10^5^) were incubated with FITC-conjugated mouse antibodies LM609 (Merck KGaA, Darmstadt, Germany), P1F6 (Merck KGaA) and A2A9/6 (Santa Cruz, Dallas, TX, USA) recognizing ανβ3, ανβ5 and αIIbβ3 integrins, respectively, for 1 h at 4 °C in the dark. To determine α5β1 levels, cell lines were incubated for 1 h at 4 °C with HA5 mouse monoclonal antibody (Merck KGaA), and then cells were washed with PBS and incubated with FITC-conjugated anti-mouse IgG (Santa Cruz, Dallas, TX, USA) for 30 min at 4 °C in the dark. Cells were subjected to FACS analysis using BD FACSDiva 8.0 software (8.0 version, BD Biosciences, Franklin Lakes, NJ, USA). At least two independent experiments were performed for each cell line and for each integrin. Results were expressed as percentage of cells positively stained for each integrin.

### 4.4. NETs Coating and Cell Adhesion Assay

A cell-free NET suspension was used to coat 24-well flat-bottom plates for cell adhesion assays. We added 5 µg of NETs in 200 µL of PBS to each well for overnight incubation at 4 °C. Diluent or conditioned medium (CM) from unstimulated neutrophil-like cells were used as negative controls. Then, multi-well plates were washed with ice-cold PBS and incubated with serum-free medium with 1% BSA for 1 h at room temperature to block non-specific adsorption. Controls for each adhesion assay included pre-treatment of isolated NET-coated plates with DNase 1 (10,000 UI/mL, Roche, Basel, Switzerland) for 15 min at room temperature to induce degradation of the DNA component of NETs and pre-incubation of cancer cells for 1 h at 4 °C with 10 µM cyclic RGD peptide (cRGD, Arg-Gly-Asp-Phe-Val) [29] for competition binding to integrins and cyclic control peptide (cCTRL, Ser-Arg-Glu-Arg-Trp) [30]. In addition, to evaluate the role of different integrins in promoting cell adhesion to NETs, cells were also pre-incubated with 45 µg/mL for 1 h at 4 °C with anti-ανβ3 LM609 (Chemicon), anti-αvβ5 P1F6 (Chemicon) or anti-α5β1 P1D6 (Abcam) blocking antibodies. Then, cells (3 × 10^5^) were added to each well in a final volume of 300 µL of serum-free medium and allowed to adhere for 1 h at 37 °C. Non-adherent cells were then removed and after a gentle washing with serum-free medium, adherent cells were detached with trypsin-ethylenediaminetetraacetic acid (EDTA) and counted. Data were expressed as a percentage of adherent cells compared to the total number of added cells. At least three independent experiments were performed in duplicates for each cell line using different NET preparations.

### 4.5. Western Blotting

Whole cell lysates were prepared using a standard protocol. Briefly, cells were washed in PBS and then lysed on ice in 100 μL of radioimmunoprecipitation assay (RIPA) lysis buffer (Tris-HCl pH 7.6, 150 mM NaCl, 1% NP-40, 1% sodium deoxycholate, 0.1% sodium dodecyl sulfate) with protease and phosphatase inhibitors (Sigma-Aldrich). The suspension was then homogenized by passaging through a 26-gauge needle and centrifuged at 13,000× *g* for 30 min at 4 °C. Western blot analysis of 40 μg of proteins from each whole cell lysate was carried out using a standard procedure [31,32]. Polyvinylidene difluoride (PVDF) membranes were probed by rabbit polyclonal antibodies (1:500) recognizing α5 (Chemicon), αIIb (Santa Cruz), β1 (Chemicon), β3 (Santa Cruz) and β5 (Santa Cruz) chains, whereas mouse monoclonal antibodies were used to probe the αv chain (clone P2W7, 1:500, Santa Cruz) and actin (Sigma; 1 μg/mL). A commercially available enhanced chemiluminescence (ECL) kit (GE Healthcare, Chicago, IL, USA) was used to reveal the reaction. At least two independent experiments were performed for each cell line and for each chain. The western blotting signal was quantified by morphodensitometric analysis using ImageJ software (NIH, Bethesda, MD, USA). Briefly, the product of the area and optical density of each band was determined and normalized to the same parameter derived from the actin control.

### 4.6. Statistical Analysis

Statistical analysis was performed using the software MedCalc for Windows, version 10.3.2.0, (MedCalc Software, Mariakerke, Belgium). Data are expressed as mean ± SE. Analysis of variance (ANOVA) followed by pairwise comparisons was used to assess differences among multiple groups. A value of *p* < 0.05 was considered statistically significant.

## Figures and Tables

**Table 1:** Expression of αvβ3, αvβ5, αIIbβ3 and α5β1 integrins in a panel of cultured tumor cell lines as determined by flow cytometry analysis.

% Positive Cells (Mean ± SE)				
Cell Line	αvβ3	αvβ5	αIIbβ3	α5β1
HT-1080	11 ± 2	96 ± 2	3 ± 2	29 ± 6
U-87 MG	79 ± 6	35 ± 7	≤1	86 ± 10
H1975	75 ± 12	80 ± 12	8 ± 1	31 ± 7
DU 145	28 ± 7	76 ± 13	10 ± 4	78 ± 5
PC-3	14 ± 10	74 ± 12	9 ± 5	8 ± 5
A-431	29 ± 5	71 ± 13	4 ± 2	4 ± 1

Results are expressed as percentage of cells positively stained with the specific antibody.

## Supplementary Materials

Supplementary materials can be found at http://www.mdpi.com/1422-0067/19/8/2350/s1.
