# Supplementary material for: Neutrophil Extracellular Traps as an Adhesion Substrate for Different Tumor Cells Expressing RGD-Binding Integrins
Source: Int J Mol Sci. 2018 Aug 9;19(8):2350. doi: 10.3390/ijms19082350 (PMC6121671; doi:10.3390/ijms19082350)
Supplement: Supplementary file 1 [file ijms-19-02350-s001.pdf]

Supplementary Materials

Neutrophil Extracellular Traps as an Adhesion Substrate for Different Tumor Cells Expressing RGD-Binding Integrins

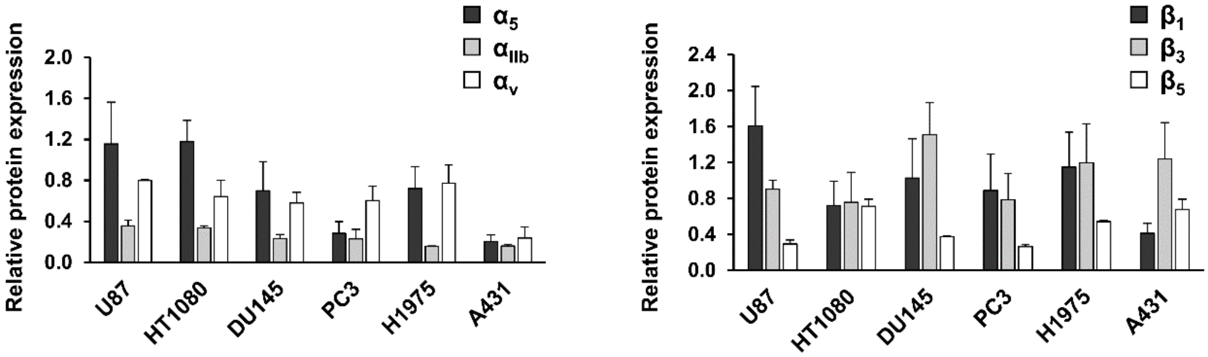

Figure S1. Levels of integrin chains by densitometric analysis of western blots. The optical density of each band was normalized to the same parameter derived from the corresponding actin control using ImageJ software. At least two independent experiment were analyzed, data were pooled and expressed as mean ± SE.
